# Self-management of falls in people with multiple sclerosis: A scoping review

**DOI:** 10.1177/02692155221128723

**Published:** 2022-09-29

**Authors:** Susanna Tuvemo Johnson, Maria Flink, Elizabeth Peterson, Kristina Gottberg, Marie Elf, Sverker Johansson, Marie Kierkegaard, Charlotte Ytterberg

**Affiliations:** 1 Department of Neurobiology, Care Sciences and Society, 27106 Karolinska Institutet, Huddinge, Sweden; 2 Academic Specialist Center, Center of Neurology, Stockholm Health Services, Stockholm, Sweden; 3 Department of Occupational Therapy, University of Illinois, Chicago, IL, USA; 4 Women’s Health and Allied Health Professionals Theme, Karolinska University Hospital, Stockholm, Sweden; 5 School of Education and Learning, Dalarna University, Falun, Sweden

**Keywords:** Behaviour, decision making, education, falls, intervention

## Abstract

**Objective:**

Falls are common in people with multiple sclerosis. There is rising interest in how the multifactorial and chronic nature of fall risk among people with multiple sclerosis can be addressed through self-management. Thus, the aims were to investigate the extent and the scope of publications on self-management of falls in people with multiple sclerosis, and to identify how the concept of self-management was defined and used.

**Data sources:**

A systematic literature search in Medline, Cochrane, Web of Science and PsycInfo was conducted to identify publications until July 2022.

**Review methods:**

Published methodological guidance was followed. Articles targeting: (1) people with multiple sclerosis, (2) falls, and (3) self-management were selected. Of 1656 records, 203 publications were assessed for eligibility, of which 173 did not meet the inclusion criteria, and 16 publications did not contain empirical data. The type of publication, study focus, and study design was extracted. If applicable, key findings, self-management tasks and skills, and the definition of self-management were extracted.

**Results:**

Fourteen original articles met all inclusion criteria. Ten articles represented six different fall prevention interventions. Three publications were randomized controlled trials. Self-management content was variable and not comprehensive in nature. None of the 14 publications included a self-management definition.

**Conclusion:**

The limited number of original articles and the even fewer intervention studies show that the research on self-management of falls in people with multiple sclerosis is in its infancy. To progress in the research area of self-management of falls, a more robust, consensus-based description of self-management frameworks and activities is needed.

## Introduction

Multiple sclerosis is the most common demyelinating disorder in high-income countries,^1^ with a global prevalence of about 36 per 100,000 inhabitants.^2^ People with multiple sclerosis may experience a variety of symptoms that are established risk factors for falls, such as impaired balance,^3,4^ mobility limitations, and impaired cognition.^3^ Behavioral, environmental and psychological influences (e.g. reduced balance confidence and fall self-efficacy) also contribute to the fall risk.^5^ The imperative to better manage fall risks is clear: people with multiple sclerosis who report a fall in the past year have an 82% probability of falling again in the following six months and a 56% probability of sustaining an injurious fall in the following six months.^6^ Injurious falls are common among ambulatory and non-ambulatory people with multiple sclerosis^7,8^ and can lead to dire consequences, including head injuries,^7^ hip fractures,^9^ and death.^10^ The consequences of inactivity after a fall can lead to deconditioning, social isolation, and lower quality of life.^11^

For people living with long-term conditions like multiple sclerosis, self-management of disease effects is essential to prevent falls during day-to-day activities.^12,13^ While there is no “gold standard” definition of self-management, the patient’s role in the day-to-day management of the chronic condition is key.^14^ Barlow et al.^15^ defined self-management as “the individual's ability to manage the symptoms, treatment, physical and psychosocial consequences, and lifestyle changes intrinsic in living with chronic conditions.” A growing body of evidence describes the benefits of self-management for people living with long-term conditions for improving self-efficacy, health behaviors, health status, and quality of life.^16^ Self-management interventions have been described as an effective part of the management of a number of chronic diseases, ranging from chronic obstructive pulmonary disease^17^ and asthma^18^ to heart failure.^19^ The relevance of self-management to multiple sclerosis is widely recognized,^20^ and there is a rising interest in using self-management approaches to mitigate the multifactorial and chronic nature of fall risks. Evidence to date indicates that efficacious self-management requires careful self-monitoring of one's condition and cognitive, behavioral, and emotional responses to coping with the medical condition.^14^ Lorig and Holman^14^ were among the first to operationalize the concept of self-management into three self-management tasks: medical management, role management, and emotional management; and six self-management skills: problem solving, decision making, resource utilization, the formation of patient–provider partnership, action planning, and self-tailoring. This conceptualization has been recognized in the multiple sclerosis self-management literature.^21,22^ To address self-management tasks and build self-management skills, self-management interventions focusing on fall risk reduction among people with multiple sclerosis often include educational components as well as opportunities to build skills (e.g. identifying fall hazards, using assertive communication strategies, developing realistic action plans).^20^

To inform self-management approaches for fall prevention among people with multiple sclerosis, a comprehensive understanding of the depth and breadth of the published literature pertaining to this area of inquiry is needed. Scoping reviews are uniquely effective in identifying gaps in the literature, especially when evidence on a given area of inquiry is just emerging.^23^ However, scoping reviews on self-management in people with multiple sclerosis are few^21,24–26^ and none focus specifically on self-management approaches to reduce fall risks. The present scoping review was undertaken to investigate the extent and the scope of publications on self-management of falls in people with multiple sclerosis, and to identify how the concept of self-management was defined and used.

## Methods

A scoping review design was used, and the study was conducted in five stages according to published methodological guidance^23,27,28^: (1) identifying the research question; (2) identifying relevant studies; (3) selecting studies; (4) charting the data; and (5) collating, summarizing, and reporting the results. We adhered to the methodological guidelines of the Preferred Reporting Items for Systematic Reviews and Meta-Analysis extension for Scoping Reviews.^29^

Identified research questions were: (1) What is the extent and nature of publications related to self-management of falls in people with multiple sclerosis? and (2) How is the concept of self-management defined and operationalized in those publications?

The research team and a research librarian developed a systematic search strategy. The search was conducted in September 2020, April 2021, and August 2022, and included publications published until July 2022. The search was limited to publications written in the English language that were published in the databases Ovid Medline, Cochrane, Web of Science, and PsycInfo. The search terms were Multiple Sclerosis, Multiple Sclerosis or Disseminated Sclerosis, Accidental falls, and Fall. Self-management was not included as a search term to allow us to include publications that did not specifically mention “self-management,” “self-care,” or other specific terms. The search strategies for each database are presented in the appendix.

The inclusion criteria were developed with the goal of targeting publications addressing (1) people with multiple sclerosis, (2) falls, and (3) self-management. Three specific inclusion criteria were applied as follows:

- the publication included descriptions related to at least one self-management task or at least one self-management skill described by Lorig and Holman,^14^ or
- the authors described their work as self-management, or
- the publication included at least one outcome pertaining to improving self-management skills, ability to engage in self-management tasks, or other behavioral changes in relation to falls.

**Figure 1.**
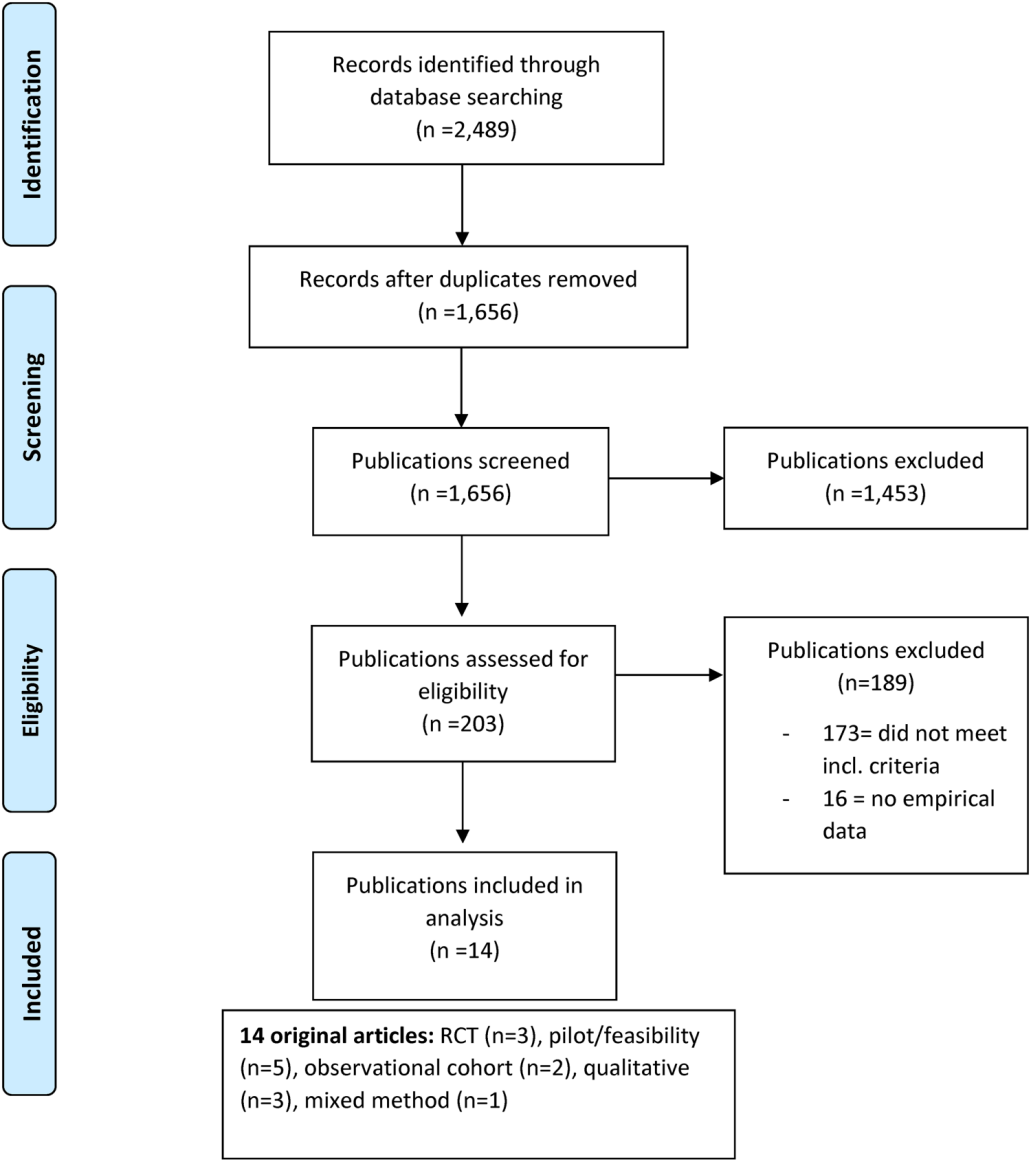
Study flow diagram.

The process of identifying relevant literature is described in Figure 1. The search yielded 1656 records. As a first step, three authors (STJ, MF and CY) independently scanned titles and abstracts whereof 1453 were excluded. In the next step, 203 publications were read in full text to identify publications that met the inclusion criteria, whereof 173 were excluded. Further, 16 publications did not include empirical data (e.g. trial registrations, conference abstracts, editorials, study protocols, and gray literature) and were therefore excluded. This assessment finally yielded 14 publications. During the selection, screening and assessment, doubts were discussed among the three authors to reach a consensus.

A data charting form was created via discussions in the research group. Year of publication, author, country of origin, type of publication, study focus, and study design were extracted from the publications. If applicable, study acronym, key findings, self-management tasks and skills, and definition of self-management were extracted. In the first step, three authors (STJ, MF and CY) extracted data. In the next step, the same three authors identified and coded which self-management tasks and/or skills according to Lorig and Holman^14^ were addressed, using a manifest-directed content analysis approach.^30^ Examples of the quotations from the included publications, and coded tasks or skills are presented in Table 1.

**Table 1.** Examples of quotations from the publications and coded task or skill.

Publication	Quotation	Coded task or skill
Peterson et al., 2009	“Participants repeatedly emphasized that their main goal was not to avoid falling, but rather to maintain their routines, fulfill their roles, and do things that were important to them.”	Role management
Kesgin et al., 2019	“Participants repeatedly mentioned their injuries and the psychological and emotional effects of falls; finding falls humiliating, embarrassing, and causing them to avoid social situations. They also mentioned feeling frustrated and helpless. It was emphasized that falls made them feel even more insecure and unsteady.”	Emotional management
Finlayson et al., 2009	“Discuss commons symptom management strategies (e.g., medications, mobility aids, physical activity).”	Medical management
Hugos et al., 2016	“FFF [Free From Falls] program is designed to increase awareness of fall prevalence and risk factors in MS, create fall-prevention strategies, develop a personal fall-prevention action plan.”	Action planning
Gunn et al., 2019	“It utilizes a number of evidence-based self-management practices, specifically group brainstorming, problem-solving and action-planning.”	Problem solving Action planning
Chanes et al., 2021	“Behavior was assessed by the survey on fall prevention strategies…, which evaluated 11 daily-life fall risk situations and the individual’s compliance with protective actions.”	Resource utilization Decision making
Gunn et al., 2018	“The nature of the relationship between the programme leader and participant was felt to be critical to achieving the balance between ‘expert’ and ‘participant.’ The overall recommendations for an approach which develops a ‘collaborative partnership.’”	Patient–provider partnership
Finlayson et al., 2009	“I plan the order of my activities in a day to manage my MS symptoms and reduce my fall risk.”	Self-tailoring

MS: multiple sclerosis.

## Results

### Extent and Nature of the Publications

In total 14 articles were included (Figure 1). The articles were published during the years 2009 to 2022 and originated in the United States of America, *n* = 8^13,31–37^; the United Kingdom, *n* = 3^38–40^; Italy *n* = 1^41^; Brazil, *n* = 1^42^; and Germany, *n* = 1.^43^ Original articles included randomized controlled trials, *n* = 3^37,41,42^; pilot/feasibility studies, *n* = 5^31,32,34,38,40^; observational cohort studies, *n* = 2^33,35^; qualitative studies, *n* = 3^13,36,43^; and mixed methods study, *n* = 1.^39^ An overview of the extent and nature of all publications including key findings, and self-management tasks and skills are presented in Table 2. Two studies specifically displayed the number of wheelchair users: Gunn, 2018^39^ one out of 15 participants, and Cattaneo, 2019^41^ 16 out of 90 participants.

**Table 2.** Extent and nature of included publications.

Author; year; country; study acronym	Study design, *n*	Study focus	Key findings/authors’ conclusions	Self-management tasks	Self-management skills
Finlayson et al; 2009; USA; Safe at home BAASE^31^	Pilot study, pre/post intervention *n* = 30 pwMS	Evaluation of outcomes of a fall risk management program	Participants reported knowledge gains and using new fall prevention and management strategies	Medical, role, emotional	Problem solving, decision-making, resource utilization, patient/healthcare provider partnership, action planning, self-tailoring
Sosnoff et al; 2015; USA^44^	Pilot feasibility RCT *n* = 34 pwMS	Evaluation of home-based exercise; education; combined exercise and education; and wait-list	Lower fall risk score in exercise groups vs non-exercise groups; no group-difference in fall prevention behavior	Medical, role	Problem solving, decision-making, resource utilization, patient/healthcare provider partnership, action planning, self-tailoring
Hugos et al; 2016; USA; FFF^33^	Retro-spective obser-vational study *n* = 134 pwMS	Evaluation of a group education and exercise program	The program improves balance confidence, balance performance, and functional mobility, and reduces falls	Role, emotional	Problem solving, decision-making, resource utilization, action planning
Cameron et al; 2022; USA; FFF^35^	RCT *n* = 96 pwMS	Evaluation of a group education and exercise program	In-person group exercise and education are not superior to written education and neurologist-initiated interventions	Not specified	Problem solving, decision-making, patient/healthcare provider partnership
Kannan et al; 2019; USA; FFFO^34^	Pilot RCT *n* = 30 pwMS	Evaluation of a web-based exercise and education fall prevention program	The program was not associated with a lower probability of falling	Emotional	Not specified
Gunn et al; 2018; UK; BRiMS^39^	Mixed-method *n* = 15 pwMS	Service users’ and providers’ views of preferred methods and delivery for a fall self-management program	The program should be tailored to the needs of pwMS; balance burden and benefit; support self-efficacy and long-term engagement; involve MS-specific expertise	Role, emotional	Problem-solving, decision-making, patient/healthcare provider partnership, action planning, self-tailoring
Gunn et al; 2019; UK; BRiMS^40^	HTA, feasibility RCT *n* = 56 pwMS	Evaluation of feasibility to finalize the design of a definitive RCT	Feasible and acceptable trial procedures Completion of daily diaries were challenging	Role, emotional	Problem-solving, decision-making, patient/healthcare provider partnership, action planning, self-tailoring
Gunn et al; 2021; UK; BRiMS^38^	Feasibility RCT *n* = 56 pwMS	Evaluation of feasibility to finalize the design of a definitive RCT	Feasible and acceptable trial procedures Refinement of methods for reporting falls is required	Role	Problem-solving, decision-making, action planning
Cattaneo et al; 2019; Italy; neuro-fall^41^	RCT *n* = 90 pwMS, stroke, and Parkinson	Test of an educational fall prevention program	No reduction in risk of falls but improved ability to carry out ADL and decreased participation restrictions	Not specified	Problem-solving, decision-making, action planning
Chanes et al; 2021; Brazil; OSE^42^	RCT *n* = 230 pwMS	Comparison of online spaced education vs educational brochure on knowledge retention and fall rate	Improvement in fall rate in both groups No significant difference	Medical, role	Problem-solving, decision-making, resource utilization, patient/healthcare provider partnership, action planning, self-tailoring
Cameron et al; 2013; USA^37^	Prospective obser-vational cohort study *n* = 58 pwMS	Comparison of fall prevention strategies among fallers and non-fallers	PwMS who fall use more fall prevention strategies than those who do not fall	Medical, role	Problem-solving, decision-making, resource utilization, patient/healthcare provider partnership, action planning, self-tailoring
Peterson et al; 2009; USA^13^	Qualitative study *n* = 6 pwMS	Description of the lived experience of falls self-efficacy during ADL	Fall self-efficacy operates as part of a larger volitional process	Role, emotional	Problem-solving, decision-making, action planning, self-tailoring
Kesgin et al; 2019; Germany^43^	Qualitative study *n* = 11 pwMS	Investigation of the views of pwMS on fall prevention programs	PwMS prefer practical, short-term programs held by healthcare professionals, in groups, not online; include safe falling techniques and exercises	Role, emotional	Problem-solving, decision-making, action planning, self-tailoring
Matsuda et al; 2021 USA^36^	Qualitative study *n* = 20 pwMS	Explore a person-centered definition of falls, circum-stances surrounding falls, and attributes facilitating program participation	“Ending up on the floor” was an agreed-upon definition for a fall; “fear of falling” was considered different from “concern about falling”; learning to fall was desirable	Not specified	Problem-solving, decision-making, patient/healthcare provider partnership, resource utilization

pwMS: people with multiple sclerosis; BAASE: safe at home behavioral attitudes activity symptoms environment; FFF/FFFO: free from falls/free from falls online; BRiMS: balance right in multiple sclerosis; HTA: health technology assessment; ADL: activities of daily living; OSE: online spaced education; IG: intervention group; CG: control group; RCT: randomized controlled trial; UK: United Kingdom; USA: United States of America.

Of the 14 included publications, 10 original articles represented six different fall prevention interventions: Safe at Home Behavioral Attitude Activity Symptoms,^31^ an exercise/education program,^32^ Free From Falls and Free From Falls Online,^33,34,37^ Balance Right in Multiple Sclerosis,^38–40^ NEUROFALL,^41^ and Online Spaced Education.^42^

*Safe at Home Behavioral Attitude Activity Symptoms* is a fall risk management program. The group-based intervention drew upon participants’ existing experiences, promoted group problem-solving, and fostered a climate of mutual support for behavioral change. It is described as a self-management program by the authors. The pre-post design used to evaluate the intervention as part of a pilot study^31^ showed a significant increase in knowledge of fall risk factors (*P* < 0.0001); significant self-efficacy improvements (*P* = 0.0016); increased knowledge and skills to manage falls and fall risk; and promoted changes in behavior to reduce personal fall risk.

*The exercise/education program* was described by the authors as being based on self-management practices. The pilot feasibility randomized controlled trial^32^ included a comparison of three different fall prevention interventions*:* (A) a home-based exercise program that targeted physiological risk factors; (B) an educational program that targeted behavioral risk factors and included problem-solving, action planning, peer-modeling, vicarious learning, social persuasion, and guided mastery; and (C) a combined exercise and educational program that targeted both physiological and behavioral risk factors for falls. The fall prevention interventions were compared to (D) a wait-list control group. Findings from the pilot feasibility randomized controlled trial revealed lower fall risk scores in exercise groups (A and C) compared to the non-exercise groups (C and D) (*P* < 0.01), but no group difference in fall prevention behavior.^32^

Both *Free From Falls* and *Free From Falls Online* are balancing exercise and education fall prevention programs delivered either face-to-face (Free From Falls) or online via webinars, and home exercises (Free From Falls Online). The three publications associated with Free From Falls and Free From Falls Online were: a retrospective observational study,^33^ a randomized controlled trial,^37^ and a pilot randomized controlled trial.^34^ Both programs aim to increase knowledge of fall risk factors, and knowledge and skills about fall prevention strategies, and are described as self-management programs. The programs feature action planning of fall prevention strategies, activities to increase fall prevention and fall management confidence, and to identify fall prevention resources in the community. Findings from a retrospective observational study showed that the Free From Falls program improved balance confidence, balance performance and functional mobility, and reduced falls; all measures improved significantly (*P* < 0.05).^33^ The Free From Falls program has been evaluated in a randomized controlled trial which did not indicate that in-person group exercise and education were superior to written education and neurologist-initiated interventions.^37^ Findings from the pilot randomized controlled trial did not indicate that the Free From Falls Online program was associated with a lower probability of falling.^34^

The *Balance Right in Multiple Sclerosis* is a home balance exercise and fall prevention education program. The three publications associated with the program included: a mixed method description of the development of the intervention,^39^ a Health Technology Assessment feasibility randomized controlled trial,^40^ and a feasibility randomized controlled trial.^38^ The Balance Right in Multiple Sclerosis is described as a self-management program aiming to improve exercise self-efficacy and to enhance participants’ knowledge and skills about fall prevention and management. It comprises educational components to increase knowledge about fall risk factors, and activities to support the development of coping strategies such as problem-solving, decision-making, action planning, and the setting and imagery of short-term goals. A collaborative, partnership approach between participants and the program leader is emphasized. The Balance Right in Multiple Sclerosis has been evaluated in a feasibility randomized controlled trial,^38^ which showed that the procedures were acceptable. Refinement of the used methods of reporting falls, i.e. prospectively reported falls diaries returned every two weeks, were required.

The *NEUROFALL* program is an educational, and mobility and balance exercise fall prevention program designed to reduce falls and increase social participation. The participants are educated about fall risk factors and strategies to both prevent falls and increase social participation. The program is not explicitly described as a self-management program by the authors but comprises problem-solving, decision-making, and action-planning activities. The program has been evaluated in a randomized controlled trial.^41^ No statistically significant between-group differences were found for the percentage number of fallers, but the program improved participants’ ability to carry out activities of daily living and decreased participation restrictions.

*Online Spaced Education* is an online education program. The primary outcomes sought through the program include knowledge retention and behavioral change to reduce falls. It is not explicitly described as a self-management program by the authors, but they state that patient education is a strategy to enhance self-management and the long-term goal of Online Spaced Education is to enhance protective behaviors related to fall risk in daily life situations. The Online Spaced Education program has been evaluated in a randomized controlled trial.^42^ Although participants in both the intervention group and the control group (who received an educational brochure) experienced reduced fall rates no significant differences between the intervention and control groups were found.

The remaining four publications included in this scoping review were: one observational cohort study^35^ which showed that people with multiple sclerosis who fall use more fall prevention strategies than those who do not fall; one phenomenological study^13^ which explored and described the lived experience of perceived self-efficacy in avoiding a fall during basic activities of daily living; and two qualitative studies which investigated: the views of people with multiple sclerosis on fall prevention programs^43^; and a person-centered definition of falls, circumstances surrounding falls, and attributes facilitating program participation.^36^

### Definition of Self-Management

None of the 14 publications included in the scoping review presented a definition of self-management. However, four publications^31,32,40,41^ referred to the Lorig and Holman framework in their intervention description or in the discussion of their findings.

### Identified Self-Management Tasks and Skills

Of the three self-management tasks, role management was highlighted most frequently, *n* = 10 publications.^13,31–35,38,41–43^ Emotional management was identified in six publications^13,31,33,34,39,43^ and medical management in four publications.^31,32,35,42^ One publication included content on all three tasks.^31^ At least two, but most often all three of the skills problem solving, decision-making, and action planning were mentioned jointly and they were the most frequently identified skills, *n* = 13.^13,31–33,35–43^ Resource utilization was the least identified skill, *n* = 6.^31–33,35,36,42^ All six self-management skills were identified in four publications.^31,32,35,42^

## Discussion

This scoping review aimed to investigate the extent and scope of publications related to self-management of falls in people with multiple sclerosis, and to identify how the concept of self-management was defined and used in the identified publications. The nature of the identified publications is consistent with an emerging field of research: 10 publications were related to six intervention programs, and only three of these publications were full-scale randomized controlled trials. Further, only two observational cohort studies and three qualitative studies were identified.

None of the publications included a definition of self-management, although several authors described their intervention as a self-management program and/or reported that their intervention included activities related to self-management. Even though there is no commonly accepted definition of self-management, there are several well-used frameworks, such as Barlow^15^ and Lorig and Holman,^14^ and more recent such as Audulv’s conceptualization of self-management for people living with neurological conditions.^12^ These frameworks are important resources that appear to be underutilized.

Four of the intervention programs referred to the Lorig and Holman framework of self-management.^31,32,40,41^ However, the program components were not consistently connected to any self-management definition or operationalization of the concept. This lack of clarity of what “self-management” entails complicates efforts to map how the intervention activities relate to established frameworks of self-management and mechanisms of impact. Evidence to date suggests that fall prevention interventions for people with multiple sclerosis that utilize self-management strategies are promising. Although the three randomized controlled trials did not show any difference between control and intervention groups regarding falls,^37,41,42^ the NEUROFALL program improved participants’ ability to carry out activities of daily living and decreased participation restrictions,^41^ and in the Online Spaced Education program both groups reduced their fall rates.^42^ The five pilot and feasibility studies^31,32,34,38,40^ also point to the potential value of self-management approaches to fall prevention for people with multiple sclerosis. In the light of this encouraging evidence, identifying the intervention content and processes that support or hinder the attainment of outcomes sought (e.g. improved fall self-efficacy, reduced fall incidence) will be instrumental to inform future intervention development efforts.

The most frequently identified self-management skills were decision-making, problem-solving, and action planning. It could be suggested that these skills are easier to embed in intervention programs as they are interrelated. The skills of resource utilization, forming of patient–provider relationship, and self-tailoring were infrequently mentioned in the publications. As people with multiple sclerosis experience diverse and interacting fall risk factors^44^ including environmental, behavioral, and physical and psychological factors, the neglect of these skills is a shortcoming. Our findings could be related to our use of a manifest-directed content analysis, which requires that only “visible, obvious components”^45^ of the text are coded. This highlights the value of both developing a consensus-based description of self-management skills for use in fall prevention programs for people with multiple sclerosis and explicitly naming those skills in intervention descriptions.

All the six intervention programs targeted primarily ambulatory people with multiple sclerosis. Multiple sclerosis is a progressive disease and approximately 25% of people with multiple sclerosis are non-ambulatory and use wheelchairs or scooters.^46,47^ While ambulatory and non-ambulatory people with multiple sclerosis share some influences on fall risk, new evidence suggests some fall risk factors for non-ambulatory people with multiple sclerosis are unique, such as performing a transfer.^48^ Therefore, it is of uttermost importance to also provide fall prevention programs for non-ambulatory people with multiple sclerosis.

A strength of the present study is that three researchers collaborated in all methodological steps^23,27,28^ and constantly discussed the progress. Another strength is the broad inclusion criteria, which increased the likelihood to cover the research area of self-management of falls for people with multiple sclerosis. In the identification of tasks and skills, we used a deductive analysis^30^ based on an established framework^14^ which allowed us to operationalize self-management despite the lack of definitions and conceptualizations in the publications.

Our review had several limitations. Our first round of review only examined the title and abstract, so relevant studies may have been missed due to, for instance, potential selection bias, which is a known limitation of the scoping review methodology.^49^ Further, as “a text always involves multiple meanings and there is always some degree of interpretation when approaching a text,”^45^ we may in our manifest-directed content analysis have misinterpreted the authors’ descriptions and either coded too few or too many as tasks or skills.

The limited number of original articles and the even fewer intervention studies show that the research on self-management of falls in people with multiple sclerosis is in its infancy. None of the publications provided a definition of self-management and rarely explicitly described self-management tasks or skills. To progress in the research area of self-management of falls for ambulatory and non-ambulatory people with multiple sclerosis, more robust, consensus-based descriptions of self-management frameworks that clearly delineate key self-management activities are needed. Naming and utilizing a shared self-management construct combined with process evaluations conducted in tandem with randomized controlled trials will inform the development of effective fall prevention interventions for people with multiple sclerosis.

**Clinical messages**

- Self-management as an approach in clinical practice to prevent falls in people with multiple sclerosis seems promising and might be a crucial factor in future multiple sclerosis health care.
- It is probably too early to implement the self-management approach in clinical guidelines due to the weak state of the evidence.

## Appendix

### Search Strategies

1. Medline

**Table:**

	Field labels.
Interface: Ovid MEDLINE(R) and Epub Ahead of Print, In-Process and Other Non-Indexed Citations and Daily. Date of Search: 17 September 2020. Number of hits: 662. Comment: In Ovid, two or more words are automatically searched as phrases; i.e. no quotation marks are needed	exp/ = exploded MeSH term. / = non exploded MeSH term. .ti,ab,kf. = title, abstract and author keywords. adjx = within x words, regardless of order. * = truncation of word for alternate endings
1. exp Multiple sclerosis/ 2. (multiple sclerosis or disseminated sclerosis).ti,ab,kf. 3. 1 or 2. 4. Accidental falls/ 5. fall*.ti,ab,kf. 6. 4 or 5. 7. 3 and 6.	

2. Cochrane Library

**Table:**

	Field labels.
Interface: Wiley Date of Search: 17 September 2020 Number of hits: 204	ti,ab,kw = title, abstract and author keywords NEAR/x = within x words, regardless of order * = truncation of word for alternate endings
#1 ("multiple sclerosis" or "disseminated sclerosis"):ab,ti,kw #2 fall*:ab,ti,kw #3 #1 AND #2	

3. Web of Science Core Collection

**Table:**

	Field labels.
Interface: Clarivate Analytics Date of Search: 17 September 2020 Number of hits: 864	TS/Topic = title, abstract, author keywords and Keywords Plus NEAR/x = within x words, regardless of order * = truncation of word for alternate endings Note: sometimes “quotation marks” are needed for single search terms to avoid automatic term mapping (lemmatization).
#1 TOPIC: ("multiple sclerosis" or "disseminated sclerosis") #2 TOPIC: (fall*) #3 #1 AND #2	

4. Psycinfo

**Table:**

	Field labels.
Interface: Ovid Date of Search: 17 September 2020 Number of hits: 168 Comment: In Ovid, two or more words are automatically searched as phrases; i.e. no quotation marks are needed	exp/ = exploded controlled term / = non exploded controlled term .ti,ab,id. = title, abstract and author keywords adjx = within x words, regardless of order * = truncation of word for alternate endings
exp Multiple Sclerosis/ (multiple sclerosis or disseminated sclerosis).ti,ab,id. 1 or 2 Falls/ fall*.ti,ab,id. 4 or 5 3 and 6	
